# Functional Insights into ANP32A-Dependent Influenza A Virus Polymerase Host Restriction

**DOI:** 10.1016/j.celrep.2017.08.061

**Published:** 2017-09-12

**Authors:** Patricia Domingues, Benjamin G. Hale

**Affiliations:** 1 Institute of Medical Virology, University of Zurich, Winterthurerstrasse 190, 8057 Zurich, Switzerland

**Keywords:** influenza virus, host range, polymerase, restriction, SUMO

## Abstract

Host restriction of influenza A virus limits pandemic emergence. The viral RNA polymerase (vPol) is an essential enzyme that must adapt for avian viruses to replicate in humans. Species differences in host ANP32A dictate adaptation: human ANP32A lacks an uncharacterized 33 amino-acid insertion that is present in avian ANP32A. Here, we uncover important contributions of host SUMOylation to vPol activity, including avANP32A function. We also identify a hydrophobic SUMO interaction motif (SIM)-like sequence unique to avANP32A that critically supports avian-signature vPol. Unrelated SIM sequences partially recapitulate this function when introduced into huANP32A. By investigating ANP32A-vPol interactions, we find that huANP32A interacts weakly with both human- and avian-signature vPols, while the hydrophobic motif of avANP32A promotes stronger interactions. Furthermore, we identify a highly acidic stretch in avANP32A that constitutes a major site of vPol interaction. Our data suggest compensatory mechanisms underlying vPol adaptation to host ANP32A independent of species-specific interactions.

## Highlights

• Host SUMOylation contributes to influenza A virus polymerase (vPol) activity
• Avian ANP32A harbors a unique hydrophobic SUMO interaction motif-like sequence
• Avian ANP32A hydrophobic motif enhances vPol interaction to overcome restriction
• Interactions between ANP32A and vPol are independent of PB2-627 identity

## Graphical Abstract

**Figure:**
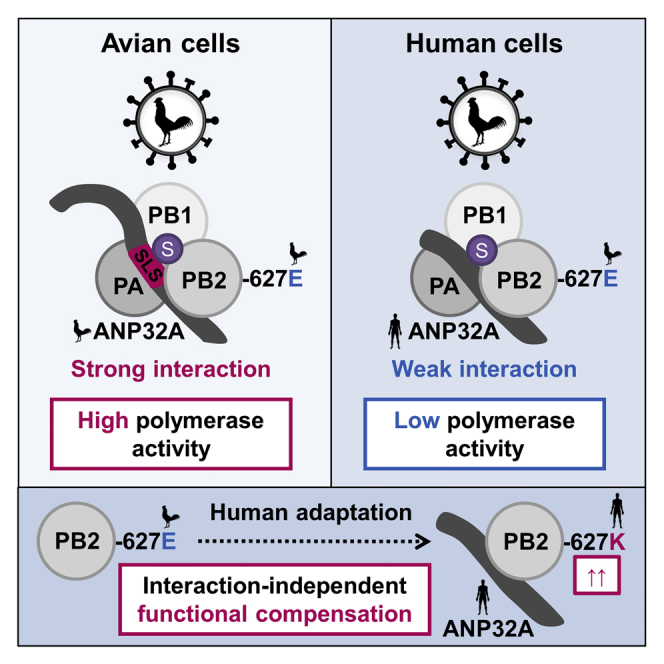

## Teaser

Species differences in cellular ANP32A dictate influenza A virus polymerase host restriction. Domingues and Hale describe the contribution of host SUMOylation to viral polymerase activity and identify a SUMO interaction motif-like sequence unique to avian ANP32A that promotes interaction with the viral polymerase and is critical for determining host range.

## Introduction

Influenza A virus (IAV) RNA-dependent RNA polymerase (vPol) is a heterotrimeric complex comprising polymerase acidic (PA), polymerase basic 1 (PB1), and polymerase basic 2 (PB2) proteins. Altogether, these subunits bind the 5′ and 3′ ends of the viral RNA genome and act with the viral nucleoprotein (NP) to form viral ribonucleoprotein complexes that complete RNA replication and transcription. A critical feature of IAV biology is that replication and transcription take place in the nucleus of infected cells, where vPol co-opts host machinery to function efficiently.

The PB2 subunit of vPol is a major contributor to host restriction (Almond, 1977), which can be determined by the identity of PB2 residue 627: PB2-627E is usually characteristic of avian-signature IAVs, often limiting replication to avian cells, while PB2-627K is an adaptive mutation characteristic of most mammalian-signature IAVs (Subbarao et al., 1993). This host-range phenotype is a consequence of host factor incompatibility and can be observed in cell-based vPol reconstitution assays using isogenic vPol pairs differing only in the identity of PB2-627. The nuclear proteins ANP32A and ANP32B, which serve functions in chromatin modification and remodeling (Reilly et al., 2014), have been identified as interactors and functional co-factors for vPol activity (Sugiyama et al., 2015). Furthermore, a species-specific difference in ANP32A was found to determine host restriction of vPol: a unique 33 amino-acid insertion in avian ANP32A (avANP32A) that is not found in human ANP32A (huANP32A), permits activity of avian-signature vPols containing PB2-627E (Long et al., 2016). One hypothesis is that the rapid selection of mammalian-signature vPol substitutions that occurs during avian IAV infection of humans may be driven by adaptation of vPol to engage the huANP32A ortholog lacking the critical insertion.

We have demonstrated that IAV infection leads to global reprogramming of host SUMOylation (Domingues et al., 2015), a ubiquitin-like modification reaction predominantly located in the cell nucleus that regulates chromatin remodeling, transcription, and DNA repair. vPol activity is a major contributor to small ubiquitin-like modifier (SUMO) redistribution, suggesting an intimate association between viral RNA replication and this post-translational modification. Here, we characterize the interplay further, uncovering a general contribution of host SUMOylation to vPol activity. We find a role for SUMO in promoting avANP32A-mediated avian-signature vPol function, which is not related to SUMO modification of avANP32A. Moreover, while we observed a weak interaction between huANP32A and vPol that is independent of PB2-627 identity, a SUMO interaction motif (SIM)-like sequence in the unique insertion of avANP32A enhances its interaction with vPol and determines restriction. These data provide insights into avANP32A-mediated enhancement of avian-signature vPols and suggest a model whereby avian-signature vPol adaptation to humans relies on overcoming consequences of weak huANP32A interaction, rather than enhancing engagement directly.

## Results

### SUMOylation Is Important for vPol Activity

**Figure 1:**
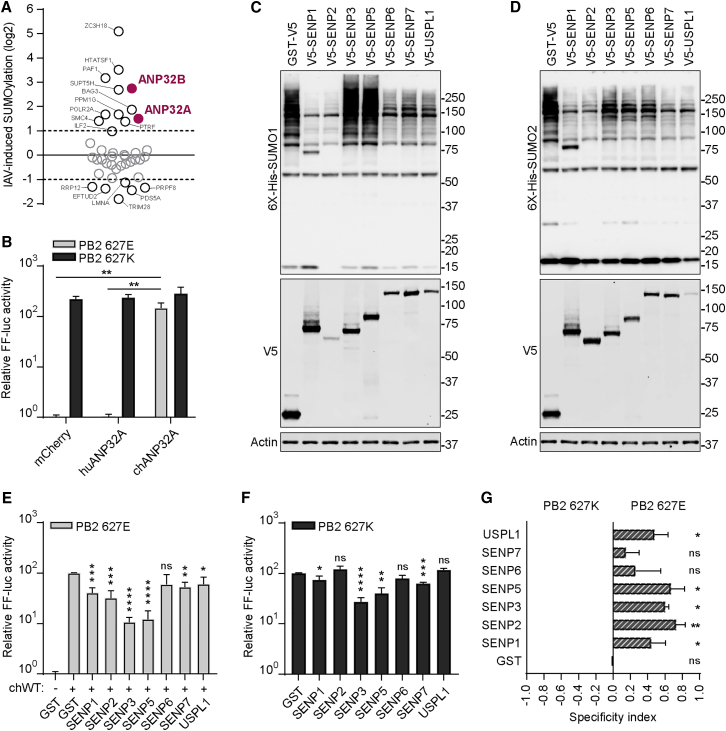
SUMOylation Is Important for vPol Activity (A) IAV-induced SUMOylation changes to vPol interactors. (B) Mini-replicon assay comparing PB2-627K and PB2-627E vPols in human cells and the impact of huANP32A and chANP32A. (C and D) Impact of SUMO proteases on SUMO1 and SUMO2 conjugates. Western blot analysis of 293T cell lysates co-transfected for 24 hr with the indicated V5-tagged SUMO protease, together with either 6X-His-tagged SUMO1 (C) or SUMO2 (D). (E) Impact of SUMO proteases on chANP32A-mediated PB2-627E vPol activity. Experiment performed as in (B), except the indicated SUMO protease was co-transfected. (F) Impact of SUMO proteases on PB2-627K vPol activity. (G) Specificity index of PB2-627E versus PB2-627K vPol activities in the presence of the indicated SUMO protease (original data from E and F; values represent 1 − E/K ± SD). In (B), (E), and (F), bars represent mean values from three independent experiments (±SD). Significance was determined by Student’s t test (^∗^p < 0.05; ^∗∗^p < 0.01; ^∗∗∗^p < 0.001; ^∗∗∗∗^p < 0.0001; NS, non-significant).

Among human host proteins that change in SUMOylation status during IAV infection (Domingues et al., 2015), we identified 18 cellular proteins previously described as vPol interactors (Bradel-Tretheway et al., 2011), including ANP32A and ANP32B (Figure 1A). To test the impact of SUMOylation on ANP32A function with respect to vPol activity, we established an ANP32A-dependent mini-replicon vPol reporter system. As expected, mini-replicon activity is highly efficient in human 293T cells when using a PB2-627K, but not PB2-627E, subunit variant (>200-fold difference) (Figure 1B). However, activity of the PB2-627E vPol can be enhanced significantly (∼150-fold) by co-expression of chicken ANP32A (chANP32A), but not huANP32A (Figure 1B). To manipulate host SUMOylation in this chANP32A-dependent system, we generated a library encompassing V5-tagged variants of all known human nuclear SUMO-specific proteases (SENP1–3, SENP5–7, and USPL1). As shown in Figures 1C and 1D, SENP1, SENP2, SENP6, and SENP7, as well as USPL1, efficiently promoted global deconjugation of SUMO1 and SUMO2 from target substrates. However, SENP3 and SENP5 exhibited specific deconjugation activity toward SUMO2 substrates. Co-transfection of each SUMO protease (except SENP6) reduced chANP32A-dependent vPol activity, with the SUMO2-specific proteases SENP3 and SENP5 reducing activity up to 10-fold (Figure 1E). Some SUMO proteases also affected activity of PB2-627K vPol complexes (Figure 1F). However, the pattern of SUMO protease-mediated inhibition of PB2-627E or PB2-627K vPol activities differed, with a trend for the PB2-627K vPol to be less sensitive to SUMO protease expression (Figure 1G). These data indicate that SUMOylation is important for efficient vPol activity but suggest that there is an additional role for SUMOylation in chANP32A-mediated avian-signature vPol function.

### chANP32A Contains a SIM-like Sequence that Promotes Avian-Signature vPol Activity

**Figure 2:**
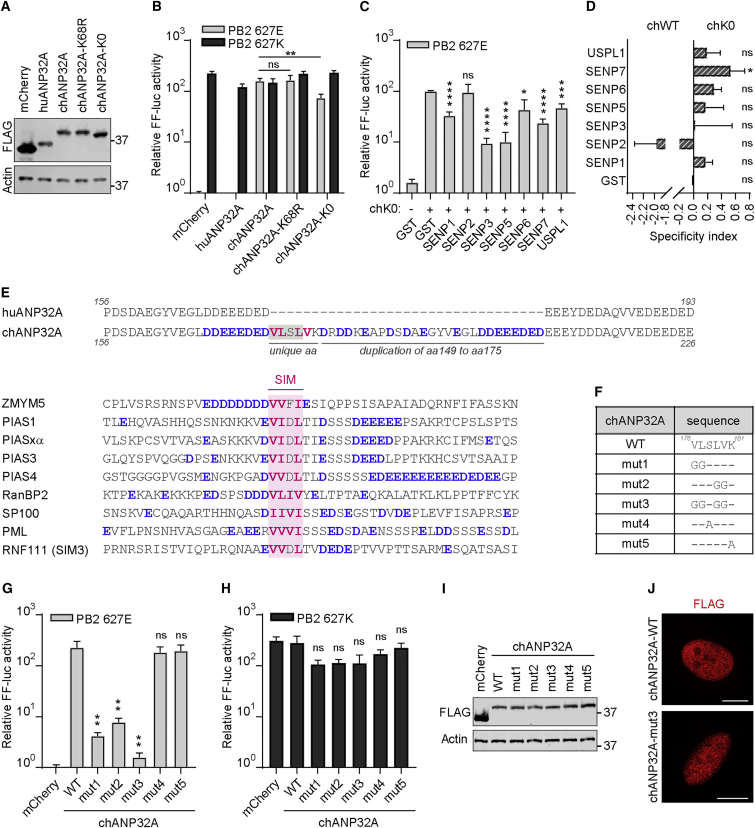
chANP32A Contains a SIM-like Sequence Important for Promoting PB2-627E vPol Activity (A) Western blot analysis of lysates from 293T cells transfected with the indicated constructs. (B) SUMOylation of chANP32A minimally affects PB2-627E vPol activity. chANP32A-K68R and chANP32A-K0 were tested for their abilities to promote PB2-627E/K vPol activities, as described in Figure 1B. (C) Impact of SUMO proteases on chANP32A-K0 function during PB2-627E vPol activity. Experiment performed as in Figure 1E. (D) Specificity index of chANP32A-WT- versus chANP32A-K0-mediated PB2-627E vPol activities in the presence of the indicated SUMO protease (original data from Figure 1E and C; values represent 1 − K0/WT ± SD). (E) (Top) Sequence analysis of the region surrounding the 33 amino-acid insertion in chANP32A. Hydrophobic residues (pink) and acidic stretches (blue), which comprise a SIM-like sequence, are highlighted. (Bottom) Alignment of bona fide SIM sequences from various human proteins. (F) Panel of chANP32A mutants generated. (G and H) Impact of chANP32A SIM-like sequence on PB2-627E (G) or PB2-627K (H) vPol activity. Experiment performed as in (B). (I) Western blot analysis of 293T cell lysates from (G). (J) Immunofluorescence analysis of the indicated FLAG-chANP32A proteins in transfected MRC5 cells. Scale bars represent 10 μm. In (B), (C), (G), and (H), bars represent mean values from three independent experiments (±SD). Significance was determined by Student’s t test (^∗^p < 0.05; ^∗∗^p < 0.01; ^∗∗∗^p < 0.001; ^∗∗∗∗^p < 0.0001; NS, non-significant).

We assessed whether direct SUMOylation of chANP32A affected its function. To this end, we generated chANP32A constructs with lysine-to-arginine loss-of-function substitutions either at the reported human ANP32B (huANP32B) SUMOylation site (residue 68; conserved in chANP32A) (Hendriks et al., 2014) or at all twenty lysine residues of the protein (termed K0). Immunoblot analysis confirmed that all constructs expressed to comparable levels (Figure 2A). chANP32A with the single K68R substitution was as efficient as wild-type (WT) at promoting avian-signature vPol activity (Figure 2B). Unexpectedly, substitution of all twenty lysine residues in chANP32A only affected its ability to promote PB2-627E vPol activity to a small degree (2-fold) (Figure 2B), which was clearly not comparable to that observed for SUMO protease co-expression. In addition, expression of most SUMO proteases still negated the vPol promoting function of the chANP32A-K0 construct to a similar extent as their effect on WT chANP32A (Figures 2C and 2D). SENP2 appeared to be more specific to WT chANP32A and had no impact on the function of chANP32A-K0, suggesting that this SUMO protease may antagonize a specific SUMOylation event on chANP32A that plays a minor role in promoting vPol activity. Nevertheless, most SUMO proteases were still highly active against chANP32A-K0-dependent vPol activity, indicating that the impact of SUMOylation on vPol activity is largely independent of ANP32A SUMOylation. These data imply that other SUMO-dependent mechanisms contribute to regulating chANP32A-dependent vPol activity.

The 33 amino-acid insertion after position 175, which is unique to chANP32A, comprises 6 novel amino-acid residues and a 27 amino-acid duplication of residues 149–175 (Figure 2E). The novel amino acids include an arrangement of hydrophobic residues (VLSLV) that resembles a SIM. Functional SIMs are often surrounded by stretches of acidic residues, which also play an important role during SIM-dependent interactions. Such an arrangement is also observed in the chANP32A protein, in which the hydrophobic residues are directly flanked to the N-terminal side by an 8 amino-acid stretch of acidic residues, and additional acidic residues and stretches are found in the C-terminal 27 amino-acid duplication (Figure 2E). Substitution of these hydrophobic residues for glycines severely affected the ability of chANP32A to support PB2-627E vPol activity, while substitutions at other non-hydrophobic positions in this motif did not alter activity (Figures 2F and 2G). When all four hydrophobic residues were substituted for glycines, the resulting chANP32A construct was effectively inactive, behaving similarly to huANP32A with respect to promoting PB2-627E containing vPol function (Figure 2G). None of the chANP32A mutant constructs negatively affected the PB2-627K vPol to a significant extent (Figure 2H), indicating a specific enhancing effect of these residues on PB2-627E. Changes to the hydrophobic patch of chANP32A did not affect either protein expression levels or intracellular localization (Figures 2I and 2J). These data indicate that the unique hydrophobic stretch of amino acids in chANP32A that resembles a SIM-like sequence is critical for promoting avian-signature vPol activity.

### Insertion of Heterologous SIM Sequences into huANP32A Partially Permits Avian-Signature vPol Activity

**Figure 3:**
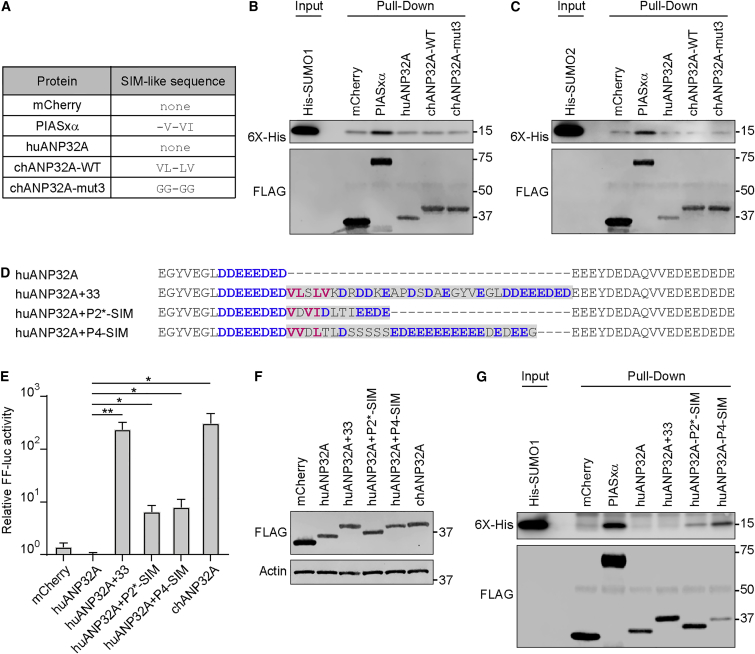
Heterologous SIM Sequences Permit huANP32A to Promote PB2-627E vPol Activity (A) Panel of constructs used and their respective SIM-like sequences. (B and C) In vitro SUMO binding assays. Recombinant 6X-His tagged SUMO1 (B) or SUMO2 (C) was added to the indicated pre-immobilized FLAG-tagged constructs. Subsequently, precipitated proteins were detected by western blot. (D) Sequence alignment of huANP32A chimeric constructs generated to include the 33 amino-acid insertion of chANP32A or heterologous SIM sequences from modified PIASxα or PIAS4. Hydrophobic residues (pink) and acidic stretches (blue), which comprise SIM-like sequences, are highlighted. (E) Impact of heterologous SIM sequences on the ability of huANP32A to promote PB2-627E vPol activity. Experiment performed as in Figure 1B. Bars represent mean values from three independent experiments (±SD). Significance was determined by Student’s t test (^∗^p < 0.05; ^∗∗^p < 0.01). (F) Western blot analysis of 293T cell lysates from (E). (G) In vitro SUMO-binding assay performed with the huANP32A chimeric constructs as described in (B).

Using in vitro SUMO binding assays, we could not detect a strong interaction between chANP32A and purified SUMO1 or SUMO2 compared with the well-characterized SIM-containing protein PIASxα (PIAS2) (Figures 3A–3C). The interaction between many bona fide SIMs and purified SUMO is known to be both transient and of very low affinity; consequently, such interactions can be difficult to detect in standard biochemical assays (Taupitz et al., 2017). We therefore used a genetic gain-of-function approach to assess the contribution of the chANP32A SIM-like sequence on avian-signature vPol activity. While huANP32A is incapable of promoting PB2-627E vPol function, a chimeric huANP32A construct harboring the unique 33 amino-acid insertion from chANP32A is fully able to support vPol activity (Figures 3D and 3E) (Long et al., 2016). We found that similar to the 33 amino acids from chANP32A, insertions of well-characterized, heterologous SIM sequences from PIAS2 or PIAS4 were able to partially enhance the ability of huANP32A to promote PB2-627E vPol activity, despite their divergent sequences and lengths (Figures 3D and 3E). All chimeric constructs expressed to similar levels (Figure 3F), and in vitro SUMO binding assays using the chimeric constructs revealed that the strong PIAS2 and PIAS4 SIMs were functional (Figure 3G). Overall, these results indicate that bona fide SIM sequences from unrelated human proteins can partially functionally replace the unique 33 amino-acid insert of chANP32A.

### chANP32A Exhibits Enhanced Interaction with vPol Complexes, which Is Mediated by the SIM-like Sequence and LCAR

**Figure 4:**
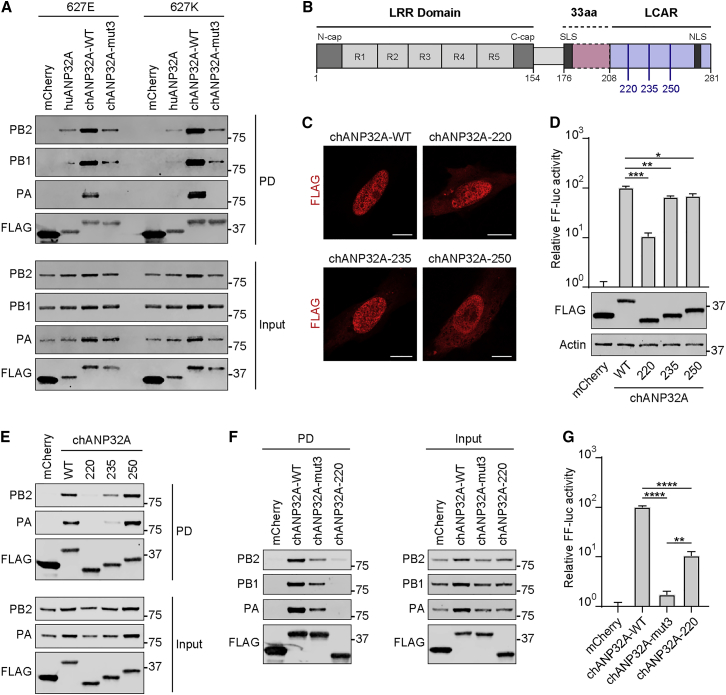
chANP32A Exhibits Enhanced Interaction with Avian- and Mammalian-Signature vPol Complexes via the SIM-like Sequence and LCAR (A) 293T cells were transfected with the indicated FLAG-tagged constructs, together with PB1, PA, and PB2 (627E or 627K). Following anti-FLAG precipitation, the indicated proteins were detected by western blot. Representative data from three independent experiments are shown. (B) Schematic representation of chANP32A. LRR, leucine-rich repeat (repeats 1–5 indicated); LCAR, low-complexity acidic region; SLS, SIM-like sequence; NLS, nuclear localization signal. (C) Immunofluorescence analysis of the indicated FLAG-chANP32A proteins in transfected MRC5 cells. Scale bars represent 10 μm. (D) (top) Impact of chANP32A LCAR truncations on PB2-627E vPol activity. Experiment performed as in Figure 1B. (bottom) Western blot analysis of 293T cell lysates from top. (E and F) Relative impact of chANP32A LCAR truncations (E) and chANP32A-mut3 (F) on PB2-627E vPol interaction. Experiments performed as in (A). Representative data from three independent experiments are shown. (G) Relative impact of chANP32A LCAR truncations and chANP32A-mut3 on PB2-627E vPol activity. Experiments performed as in Figure 1B. In (D) and (G), bars represent mean values from three independent experiments (±SD). Significance was determined by Student’s t test (^∗^p < 0.05; ^∗∗^p < 0.01; ^∗∗∗^p < 0.001; ^∗∗∗∗^p < 0.0001).

huANP32A was previously shown to interact with the human-adapted vPol to regulate viral genome synthesis (Sugiyama et al., 2015). We therefore tested the hypothesis that chANP32A is able to support PB2-627E vPol activity by exhibiting a higher affinity toward this avian-signature complex than the huANP32A ortholog. As shown in Figure 4A, we confirmed that huANP32A can weakly co-precipitate the PB2-627E vPol complex and further found that chANP32A interacts more efficiently with this complex. Surprisingly, this enhanced interaction of chANP32A with vPol was not specific to PB2-627E, because chANP32A also showed greater interaction with the human-signature PB2-627K vPol (Figure 4A). Similarly, we found that the weaker co-precipitation of vPol by huANP32A is independent of PB2-627 identity. The enhanced interaction of chANP32A with the vPol complex is mediated by the SIM-like sequence in the unique 33 amino-acid insertion of chANP32A, because substitution of the key hydrophobic residues for glycines severely affected the ability of chANP32A to precipitate vPol (Figure 4A). These results reveal that chANP32A exhibits a greater propensity than huANP32A to interact with vPol in co-transfected cells but that this does not correlate with the identity of PB2-627. Furthermore, the requirement of the SIM-like sequence in chANP32A for mediating this enhanced interaction suggests that an additional SUMO-modified factor could stabilize the chANP32A-vPol complex.

To better understand the chANP32A-vPol interaction, we constructed a series of additional chANP32A mutants and examined their ability to co-precipitate the viral complex and support vPol activity. chANP32A, by homology to huANP32A, consists of an N-terminal leucine-rich repeat (LRR) domain flanking the 33 amino-acid insert that contains the SIM-like sequence. In addition, the C-terminal part of chANP32A comprises a low-complexity acidic region (LCAR), with the only recognizable motif being a short stretch of basic amino-acid residues near the C terminus that make up a nuclear localization signal (NLS) (Figure 4B). Because the ANP32A LRR domain is highly structured, and small deletions likely lead to global unfolding of this domain, we focused our mutational analysis on the LCAR, producing a series of C-terminal deletions. All mutant constructs expressed equally well, suggesting that they were stable, and as expected, removal of the NLS prevented absolute retention of chANP32A in the nucleus so that a more even nuclear-cytoplasmic distribution was observable (Figure 4C). Deletion of the C-terminal 46 residues of chANP32A, which includes the NLS, had a relatively minor impact on the ability of chANP32A to promote PB2-627E vPol function. In contrast, a chANP32A mutant construct lacking 61 residues (chANP32A-220) was more severely compromised in promoting vPol activity (Figure 4D). Interaction analyses revealed that chANP32A-220 was the most defective at co-precipitating vPol, although binding of the chANP32A-235 construct was also affected (Figure 4E). These data define chANP32A residues 221–235 as major determinants of vPol binding and contributors to PB2-627E vPol activity. This particular region is an almost contiguous stretch of 14 acidic residues, suggesting that this association with vPol is mediated by electrostatic interactions. Side-by-side comparisons revealed that the vPol interaction mediated by chANP32A residues 221–235 is more essential than that mediated by the SIM-like sequence (Figure 4F), even though mutation of the SIM-like sequence has a greater impact on reducing the ability of chANP32A to promote PB2-627E vPol function (Figure 4G). These data suggest that although strong interaction of chANP32A to vPol is important for overcoming PB2-627E host restriction, the most critical determinant of activity is integrity of the SIM-like sequence.

## Discussion

Herein, we show that host SUMOylation plays an important role during vPol activity. Expanding on this, we provide evidence that a SUMO-dependent function may contribute to the ability of avANP32A to interact with, and promote activity of, avian-signature vPols. We identified a critical role for a hydrophobic motif in the unique 33 amino-acid insertion of avANP32A that, together with surrounding acidic residue stretches, shows a striking resemblance to SIM-like sequences that normally mediate non-covalent interactions with SUMO. Although we were unable to show that this hydrophobic sequence in avANP32A can directly bind purified SUMO1 or SUMO2 in vitro, we used genetic gain-of-function experiments to highlight that unrelated heterologous SIM sequences can to some extent functionally replace the SIM-like sequence in avANP32A. The lack of full complementation by these heterologous SIM sequences may be due to unknown differences with certain unique features of the avANP32A SIM-like sequence that dictate SUMO paralog specificity or binding orientation. However, our observations suggest that if the hydrophobic motif in avANP32A constitutes a functional SIM, then the direct interaction with isolated SUMO is probably weak, a property also exhibited by many bona fide SIM-containing proteins (Taupitz et al., 2017). It may be that the SIM-like sequence in avANP32A only shows affinity for SUMO that is conjugated to a particular host or viral factor. Host factors previously implicated in contributing toward vPol restriction, such as importin family members, RIG-I, TUFM, and DDX17 (Bortz et al., 2011, Gabriel et al., 2011, Kuo et al., 2017, Weber et al., 2015), have also been reported to be SUMO modified (Hendriks et al., 2014, Hu et al., 2017, Lamoliatte et al., 2014, Mooney et al., 2010, Wen et al., 2014). We speculate that avANP32A stably engages with vPol via a SUMO-modified viral or host factor.

The huANP32A protein was previously shown to interact with a trimeric human-adapted vPol complex (Sugiyama et al., 2015). Here, we made the surprising finding that huANP32A interacts weakly with both human- and avian-signature vPols and that avANP32A exhibits an enhanced interaction with the vPol complex (also independent of PB2-627 identity) that is dependent upon the hydrophobic SIM-like sequence. We additionally identified a highly acidic stretch of 15 amino acids in the ANP32A LCAR (residues 221–235) that appear critical for interaction with vPol yet are not as important as the SIM-like sequence for promoting vPol activity. Given these observations, it seems unlikely that selection of the viral PB2-627K adaptive mutation in human cells is directly due to favored species-specific binding toward the shorter mammalian ANP32A protein. Nevertheless, it is clear that a functional correlation exists between species differences in ANP32A length and vPol host adaptation (Long et al., 2016). Thus, our data may support a working model whereby the mammalian-adapted vPol (e.g., PB2-627K) arises to compensate for weak or transient recruitment of huANP32A, perhaps by increasing efficiency of huANP32A use, rather than by enhancing binding. This would also suggest that avian-adapted PB2 is inefficient at using ANP32A, but this is compensated in avian cells by the strong and stable interaction of avANP32A with vPol, potentially via a SUMO-modified factor, which permits a longer interplay between PB2-627E and ANP32A. Although hypothetical, such a model would agree with the breakthrough finding that vPol must adapt to host-specific ANP32A (Long et al., 2016) while integrating previous models detailing host adaptation mediated by vPol strategies to increase functional efficiency (Paterson et al., 2014, Rameix-Welti et al., 2009).

In summary, we provide evidence that SUMOylation contributes to vPol activity and that a unique hydrophobic SIM-like sequence in avANP32A promotes interaction with vPol and is critical to overcome vPol host restriction mediated by avian-signature PB2-627E. Our work contributes to understanding of the interplay between mammalian and avian vPol complexes and a key host-range determinant.

## Experimental Procedures

### Cells

293T cells were maintained in DMEM supplemented with 10% (v/v) fetal calf serum, 100 units/mL of penicillin, and 100 μg/mL of streptomycin. MRC5 cells were maintained in Eagle’s minimal essential medium (EMEM) supplemented with 10% (v/v) fetal calf serum, 100 units/mL of penicillin, 100 μg/mL of streptomycin, 2 mM L-glutamine, and 1% (v/v) non-essential amino acids.

### Plasmids

Sequences of human SENP1–3, SENP5–7, and USPL1 were PCR amplified from existing plasmids (gifts from Edward Yeh or Wade Harper [Addgene plasmids #17357, #18047, #18048, #18053, #18065, #42886, and #22607]) (Bawa-Khalfe et al., 2012, Cheng et al., 2007, Dou et al., 2010, Gong and Yeh, 2006, Kang et al., 2010, Sowa et al., 2009) and ligated in frame into a modified pCAGGS vector to express with N-terminal V5 tags. Codon-optimized sequences of huANP32A-WT, chANP32A-WT, chANP32A-K0, huANP32A+33, huANP32A+P2^∗^-SIM, and huANP32A+P4-SIM were generated using the GeneArt gene synthesis service (Thermo Fisher Scientific) and ligated in frame into p3xFLAG-CMV-7.1 (Sigma-Aldrich) so as to express with N-terminal FLAG tags. The plasmid expressing FLAG-hPIASxα was a gift from Ke Shuai (Addgene plasmid #15209) (Arora et al., 2003). pCAGGS-GST-V5, pLVX-6X-His-SUMO1, and pCAGGS-6X-His-SUMO2 plasmids were generated by standard methods. The expression plasmid for FLAG-tagged mCherry has been described previously (Domingues et al., 2015). Indicated additional gene variants, as well as any required nucleotide corrections, were generated by Quikchange II XL Site-Directed Mutagenesis (Agilent Technologies). New constructs were authenticated by DNA sequencing. All plasmid transfections were performed using Fugene HD (Promega) at a 1:3 DNA:transfection reagent ratio.

### Mini-replicon Assays

2 × 10^5^ 293T cells were co-transfected with pPolI-A/WSN/33 (WSN)-FFluc (negative-sense viral-like *Firefly* luciferase reporter), pRL-SV40 (constitutive *Renilla* luciferase reporter) (Promega), and pCAGGS expression vectors encoding WSN PA, PB1, and PB2 (627E or 627K) and NP (at a ratio of 1:2:2:4:2 for PA:PB1:PB2:NP:reporter), together with the indicated amount of the plasmids of interest. pRL-SV40 was used as a normalization control. 24 hr later, cells were harvested and luciferase activity was measured using the Dual-Glo Luciferase Assay system (Promega).

### Co-immunoprecipitation Assays

For SUMO-binding assays, 293T cells (∼2 × 10^6^ in a 25 cm^2^ flask) were transfected for 48 hr with 3 μg of the indicated FLAG-tagged construct before lysis in buffer A (50 mM HEPES [pH 7.5], 100 mM NaCl, 50 mM KCl, 0.25% NP-40, and 1 mM DTT), supplemented with 5 mM β-glycerophosphate, 1 mM sodium orthovanadate, 25 mM NaF, and a cOmplete Mini EDTA-free protease inhibitor cocktail (Roche). Following sonication and centrifugation (13,000 rpm, 20 min, and 4°C), the soluble lysate fractions were incubated end over end at 4°C for 2 hr with anti-FLAG M2 Affinity Agarose beads (Sigma). Precipitated immune complexes were washed extensively in buffer A before recombinant human His-SUMO1 (UL-715, Boston Biochem) or His-SUMO2 (UL-752, Boston Biochem) was added at a concentration of 2 μg/mL and reactions were incubated end over end at 4°C for 16 hr. Protein-bead complexes were further washed in buffer A before elution in 2× urea disruption buffer (6 M urea, 2 M β-mercaptoethanol, and 4% SDS).

For vPol interaction assays, 293T cells (∼2 × 10^6^ in a 25 cm^2^ flask) were co-transfected with 3 μg of the indicated FLAG-tagged construct, together with pCAGGS vectors expressing PA (0.5 μg), PB1 (1.5 μg), and PB2 (1.5 μg). 48 hr later, cells were lysed in buffer A supplemented with 20 mM iodoacetamide (IAA), 5 mM β-glycerophosphate, 1 mM sodium orthovanadate, 25 mM NaF, and a cOmplete Mini EDTA-free protease inhibitor cocktail (Roche). Following sonication and centrifugation (as earlier), the soluble lysate fractions were incubated end over end at 4°C for 16 hr with anti-FLAG beads. Precipitated immune complexes were further washed in buffer A supplemented with 20 mM IAA and were eluted from the beads using 2× urea disruption buffer.

### Western Blot Analyses

Samples were lysed 1:1 in 2× urea disruption buffer, sonicated, and then boiled for 10 min before SDS-PAGE on NuPAGE Novex 4%–12% Bis-Tris gradient gels (Thermo Fisher Scientific). Proteins were detected by western blotting after transfer to polyvinylidene fluoride (PVDF) or nitrocellulose membranes using the following antibodies: IAV-PB1 (cc11), IAV-PB2 (polyclonal anti-serum), IAV-PA (9016) (gifts from Silke Stertz), FLAG M2 (Sigma, F1804), 6X-His tag (Abcam, ab18184), actin (Sigma, A2103), or V5 tag (Bio-Rad, MCA1360).

### Immunofluorescence Imaging

MRC5 cells transfected with the indicated FLAG-tagged constructs for 24 hr were fixed, permeabilized, and stained with anti-FLAG M2 antibody (Sigma, F1804) as described (Domingues et al., 2015). Images were taken on a Leica SP5 confocal microscope.

### Statistics

Statistical analyses were performed using an unpaired two-tailed Student’s t test. The p values for significance are stated in the figure legends.

## Author Contributions

Conceptualization and Methodology, P.D. and B.G.H.; Investigation, Visualization, and Writing – Original Draft, P.D.; Funding Acquisition, Supervision, and Writing – Review and Editing, B.G.H.
